# Fibrosis-related miRNAs as serum biomarkers for pancreatic ductal adenocarcinoma

**DOI:** 10.18632/oncotarget.23377

**Published:** 2017-12-17

**Authors:** Rei Suzuki, Hiroyuki Asama, Yuichi Waragai, Tadayuki Takagi, Takuto Hikichi, Mitsuru Sugimoto, Naoki Konno, Ko Watanabe, Jun Nakamura, Hitomi Kikuchi, Yuki Sato, Shigeru Marubashi, Atsushi Masamune, Hiromasa Ohira

**Affiliations:** ^1^ Department of Gastroenterology, Fukushima Medical University School of Medicine, Fukushima, Japan; ^2^ Department of Endoscopy, Fukushima Medical University Hospital, Fukushima, Japan; ^3^ Department of Hepato-Biliary-Pancreatic and Transplant Surgery, Fukushima Medical University School of Medicine, Fukushima, Japan; ^4^ Division of Gastroenterology, Tohoku University Graduate School of Medicine, Sendai, Japan

**Keywords:** Pancreatic cancer, microRNA, fibrosis, digital PCR, chemotherapy

## Abstract

We investigated whether serum microRNAs (miRNAs) could be diagnostic or prognostic markers in pancreatic ductal adenocarcinoma (PDAC). We first identified miRNAs showing altered expression in human pancreatic stellate cells (hPSCs) co-cultured with PDAC cells (Panc-1 and BxPC-3) as compared to hPSCs cultured alone. Among the miRNAs with altered expression, let-7d exhibited reduced expression in an *in silico* analysis of The Cancer Genome Atlas data. Inhibition of let-7d resulted in enhanced expression of fibrosis-related genes. We extracted serum miRNA from 45 PDAC patients and 42 healthy controls and quantified expression let-7d using digital PCR. Based on the level of let-7d expression, we were able to distinguish between PDAC patients and controls. Additionally, reduced let-7d expression correlated with poor overall survival. Thus, fibrosis-related miRNAs may be serum biomarkers for PDAC.

## INTRODUCTION

Pancreatic ductal adenocarcinoma (PDAC) is one of the most lethal malignancies worldwide [1]. Surgical resection is the only curative treatment for PDAC. However, less than 20 percent of patients are candidates for pancreatectomy because of advanced disease stage at presentation [2, 3]. The median survival time of patients with unresectable PDAC who are treated with newly established chemotherapy regimens (e.g., FOLFIRINOX and gemcitabine plus nab-paclitaxel) is approximately 1 year [4–7]. Therefore, the development of less invasive diagnostic methods such as PDAC-specific blood biomarkers, is important in order to detect early-stage disease and improve patient prognosis.

The desmoplastic reaction is characterized by a dense concentration of extracellular matrix proteins, activated pancreatic stellate cells (PSCs), and immune cells surrounding tumor cells [8]. It contributes to tumor development and has been shown to inhibit drug penetration and uptake in PDAC and other malignancies [9–11]. Although various proteins associated with the desmoplastic reaction have been identified, they have not proven to be useful blood markers [12–14]. MicroRNAs (miRNAs) are small, noncoding RNAs (18–25 nucleotides in length) that regulate gene expression at the post-transcriptional level by promoting degradation of mRNAs or by blocking mRNA translation. Therefore, they play essential roles in various biological processes. Recently, circulating miRNAs have been demonstrated to have diagnostic potential as blood biomarkers for various tumors [15–17]. Circulating miRNAs can be detected with real-time PCR, which typically requires standard curve generation or normalization to a spike-in control [18]. In contrast, digital PCR eliminates the need for normalization and is highly reproducible [18–20].

In this study, we used digital PCR to identify PDAC-specific circulating miRNAs and evaluated whether they could be used to detect PDAC. We analyzed differential miRNA expression in human pancreatic stellate cells (hPSCs) co-cultured with PDAC cells mimicking cancer-associated fibroblasts. Additionally, the levels of PDAC-specific miRNAs were analyzed in serum from PDAC patients.

## RESULTS

### Altered expression of fibrosis-related genes and miRNAs in co-cultures of hPSCs and PDAC cells

**Figure 1:**
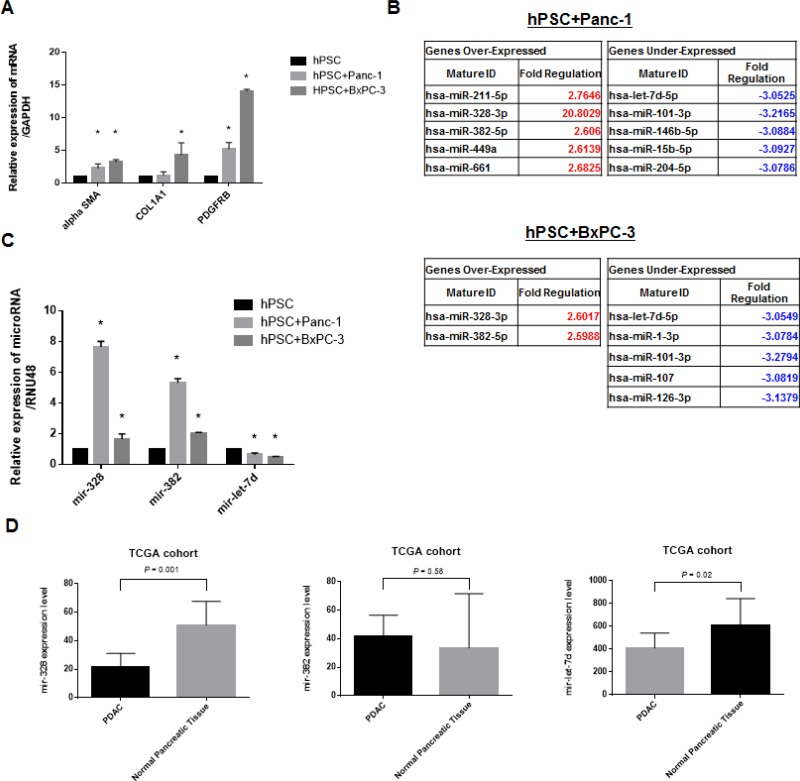
Altered expression of fibrosis-related genes and miRNAs in co-culture experiments (**A**) Increased expression of α-SMA, PDGFRβ, and COL1A1 in hPSC21-S/T (hPSCs) after 72 hours of co-culture with PDAC cells. (**B**) MiRNA profiling assays demonstrating altered expression of several miRNAs including mir-328, mir-382, and mir-let-7d in hPSCs co-cultured with PDAC cells compared to hPSCs alone. (**C**) Real-time PCR demonstrating altered miRNA expression in the co-cultures. (**D**) Analysis of differences in miRNA expression between PDAC (*n* = 183) and normal tissue (*n* = 4) was evaluated by real-time PCR. Only let-7d showed consistently reduced expression. The opposite results were observed for mir-328, and no changes in mir-382 expression were observed. ^*^P < 0.05.

We observed higher expression of three fibrosis-related genes (α-SMA, PDGFRβ, and COL1A1) in hPSCs 72 hours after co-culture with PDAC cells (Figure 1A). MiRNA profiling assays revealed that several miRNAs were differentially expressed in hPSCs co-cultured with PDAC cells compared to hPSCs alone (Figure 1B). We validated the expression of three miRNAs (mir-328, mir-382, and let-7d) by real-time PCR. An increase in mir-328 and mir-382, and a decrease in let-7d expression, were observed in hPSCs co-cultured with Panc-1 or BxPC-3 cells (Figure 1C and Supplementary Figure 1). We next investigated the expression of these miRNAs in pancreatic cancer compared to normal tissue using data from The Cancer Genome Atlas (TCGA). Let-7d expression was consistently reduced in pancreatic cancer compared to normal tissue. However, altered mir-328 and mir-382 expression was not observed (Figure 1D).

### Inhibition of let-7d enhances the expression of fibrosis-related genes via the TGF-β pathway

**Figure 2:**
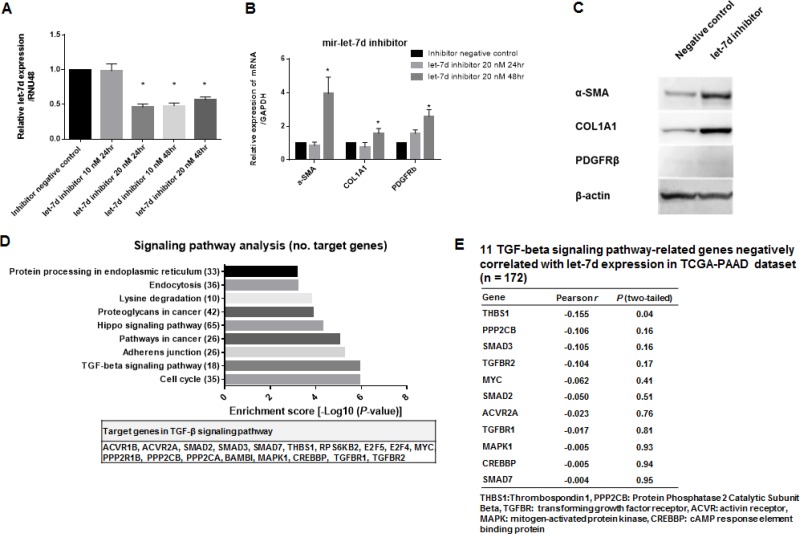
Inhibition of let-7d enhances the expression of fibrosis-related genes via the TGF-β pathway (**A**) Decreased let-7d expression following transient transfection of the cells with the let-7d inhibitor. (**B**–**C**) Decreased let-7d levels resulted in enhanced expression of α-SMA, PDGFRβ, and COL1A1. (**D**) Pathway enrichment analysis revealed that let-7d could target 30 different signaling pathways. The top nine pathways with the highest enrichment scores (e.g. the TGF-β pathway) are shown. (**E**) A negative correlation between THBS1 and let-7d expression was observed in the mRNA and miRNA analysis of TCGA data (Pearson *r* = –0.155, *P* = 0.04).

A time- and concentration-dependent decrease in let-7d expression was observed following transient transfection of cells with a let-7d inhibitor (Figure 2A). Based on these results, we selected a concentration of 20 nM of the let-7d inhibitor for all subsequent analyses. Gene expression assays revealed that inhibition of let-7d expression resulted in enhanced expression of fibrosis-related genes (Figure 2B–2C). To identify the targets of let-7d, we used the DIANA-miRPath software. We found that let-7d targeted 18 genes in the TGF-β pathway (Figure 2D). Integrated mRNA and miRNA analysis using TCGA data indicated that thrombospondin 1 (THBS1) expression was negatively correlated with let-7d expression (Pearson *r* = –0.155, *P* = 0.04) (Figure 2E).

### Serum miRNA levels could be diagnostic and prognostic markers in PDAC

**Figure 3:**
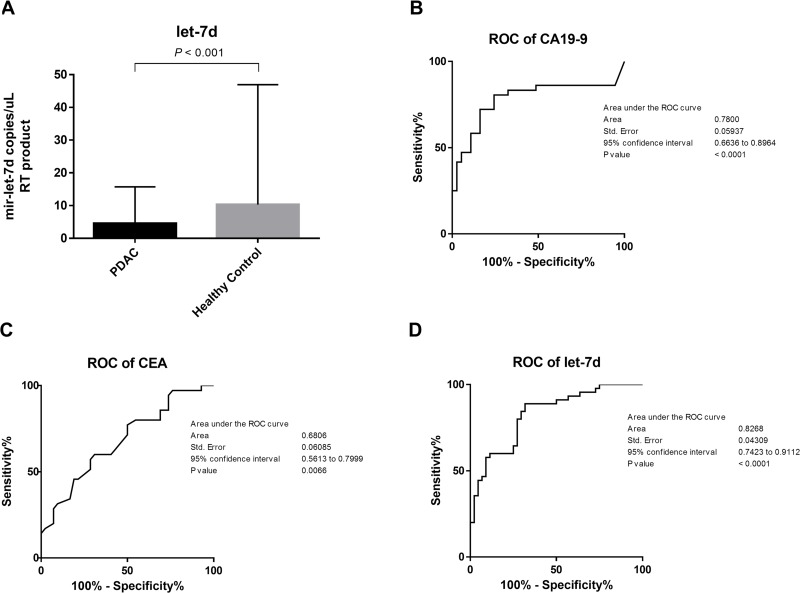
Differentiation between PDAC patients and healthy controls using serum miRNAs (**A**) The levels of CA 19–9 and CEA in serum were higher in PDAC patients compared to controls (271.3 vs. 10.5 U/mL, *P* < 0.0001 and 3.1 vs. 2.0 ng/mL, *P* = 0.006, respectively). Serum mir-let-7d expression was lower in PDAC patients compared to controls (4.5 vs. 10.3 copies/μL, *P* < 0.001). (**B–D**) ROC analysis demonstrated that these three markers could discriminate between PDAC patients and controls with an AUC of 0.68 for CEA (95% CI: 0.56 to 0.79), 0.78 for CA 19-9 (95% CI: 0.66 to 0.89), and 0.83 for mir-let-7d (95% CI: 0.74 to 0.91).

Serum carbohydrate antigen 19-9 (CA 19-9) and carcinoembryonic antigen (CEA) levels were higher in PDAC compared to control samples (271.3 vs. 10.5 U/mL, *P* < 0.0001 and 3.1 vs. 2.0 ng/mL, *P* = 0.006, respectively; Table 1). In contrast, serum mir-let-7d expression was lower in PDAC compared to control samples (4.5 vs. 10.3 copies/μL, *P* < 0.001) (Figure 3A). Receiver operating characteristic (ROC) analysis showed that these three markers could discriminate between cancer patients and controls, with an area under the curve [AUC] of 0.68 for CEA (95% confidence interval [CI]: 0.56 to 0.79), 0.78 for CA 19-9 (95% CI: 0.66 to 0.89), and 0.83 for mir-let-7d (95% CI: 0.74 to 0.91). At the cutoff value for CA 19-9 (46.5 U/mL), the sensitivity and the specificity were 80.5% and 75.6%, respectively. At the cutoff value for CEA (2.25 ng/mL), the sensitivity and the specificity were 60.0% and 69.0%, respectively. Finally, at the cutoff value for mir-let-7d (7.8 copies/μL), the sensitivity and the specificity were 88.9% and 68.2%, respectively (Figure 3B–3D).

**Table 1:** Clinical characteristics of patients

No. (%) of patients and healthy controls (*n* = 87)	
Pancreatic cancer	45 (51.7)
Sex, male/female	28 (62.2)/17 (37.8)
Age, median (range), y	67.0 (45.0–89.0)
Resection of tumors, yes/no	8 (17.7)/37 (82.3)
Cancer Stage (UICC)	
IA	1 (1.8)
IIA	5 (10.8)
IIB	2 (4.4)
III	13 (28.9)
IV	24 (53.3)
Serum CA 19-9, median (range), U/mL	271.3 (2.0–33,739)
Serum CEA, median (range), ng/mL	3.1 (1.2–82.9)
Non-tumor controls	42 (48.2)
Chronic pancreatitis	18 (41.8)
Biliary stone	20 (47.6)
Others	4 (9.5)
Sex, male/female	26 (61.9)/16 (38.0)
Age, median (range), y	72.9 (47.0–94.0)
Serum CA 19-9, median (range), U/mL	10.5 (2.0–1929)
Serum CEA, median (range), ng/mL	2.0 (0.6–14.4)

UICC: Union for International Cancer Control.

**Figure 4:**
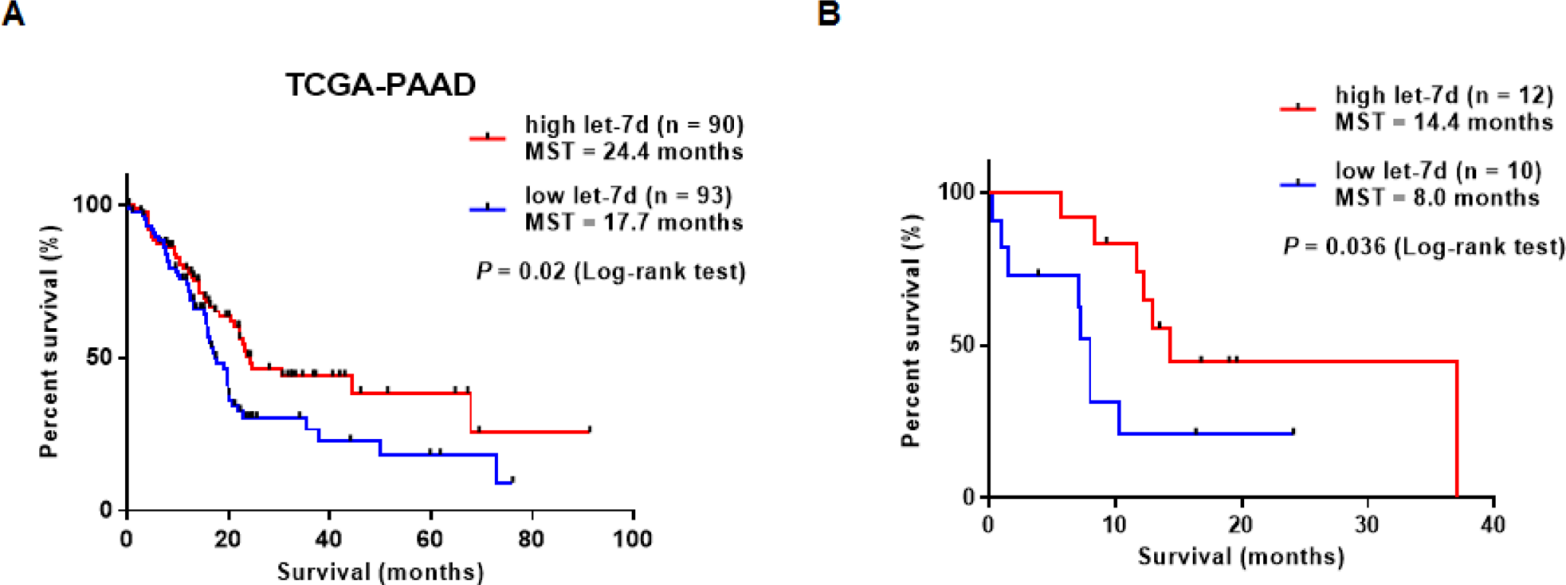
Survival analysis of PDAC patients with differential let-7d expression (**A**) Analysis of TCGA data demonstrated that the overall survival of PDAC patients with high let-7d expression was longer than that of patients with low let-7d expression (median overall survival: 24.4 vs. 17.7 months (log-rank test; *P* = 0.02). (**B**) Serum let-7d expression could predict the response of PDAC patients to gemcitabine-based chemotherapy.

We performed survival analyses of 183 PDAC patients retrieved from TCGA database. Patients were divided into high let-7d (*n* = 90) and low let-7d expression groups (*n* = 93). The overall survival of patients with high serum let-7d expression was longer than that of patients with low let-7d expression (median overall survival period: 24.4 vs. 17.7 months, log-rank test; *P* = 0.02) (Figure 4A). We also compared the overall survival of 22 patients with high or low serum let-7d expression who received gemcitabine-based chemotherapy. We divided the patients according to the median expression value for each miRNA (5.9 copies/μL for let-7d). In this analysis, we found that let-7d expression could identify patients with poor survival (undefined vs. 9.3 months, log-rank test; *P* = 0.04) (Figure 4B).

### Correlation between intra-tumor fibrosis on computed tomography and the levels of serum miRNAs

**Figure 5:**
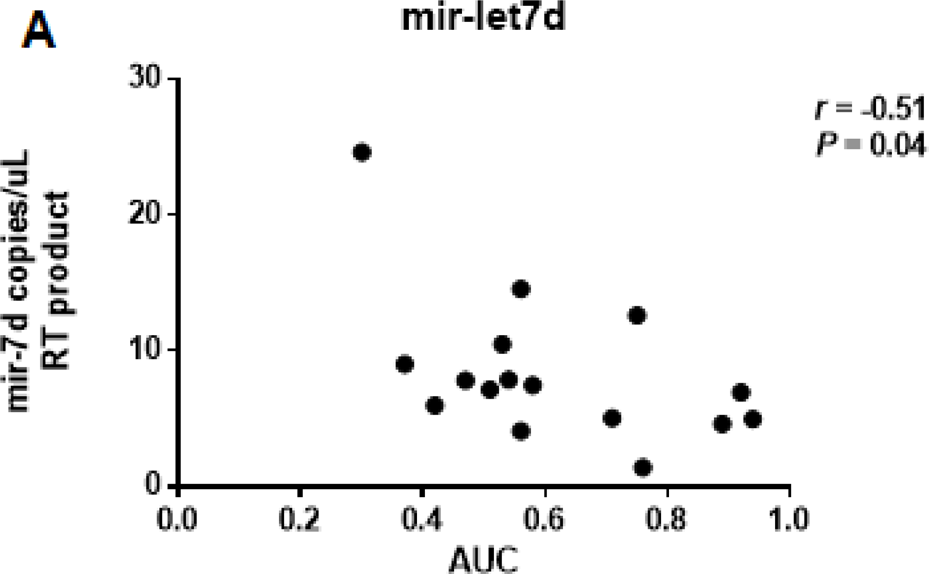
Correlation between the CT-derived normalized AUC and the expression of serum let-7d The normalized AUC was calculated with a median value of 0.56 (range: 0.30 and 0.94). Let-7d was negatively correlated with the AUC (*r* = –0.51, *P* = 0.04).

Computed tomography (CT) data was available for 16 PDAC patients. The normalized AUC was calculated with a median value of 0.56 (range: 0.30 and 0.94). Let-7d levels were negatively correlated with the normalized AUC (*r* = –0.51, *P* = 0.04) (Figure 5).

## DISCUSSION

We identified miRNAs that were associated with PDAC-induced hPSC activation and fibrosis. These miRNAs may have diagnostic and prognostic potential in PDAC. Our data indicate reduced serum let-7d levels are associated with PDAC-induced hPSC activation.

The interaction between PDAC cells and PSCs has been widely studied. Co-culture experiments have revealed that many proteins, mRNAs, and miRNAs are differentially expressed in PDAC and can promote tumor cell invasion and migration [21–23]. Among these differentially expressed factors, miRNAs are of particular interest because they regulate cell-cell interactions.

Takikawa *et al*. demonstrated that hPSC-derived exosomes contained various miRNAs including mir-21-5p and that increased expression of chemokine ligands 1 and 2 in pancreatic cancer cells enhanced tumor cell proliferation and migration [23]. Ali *et al*. demonstrated altered expression of mir-21 and mir-221 in hPSCs co-cultured with PDAC cells, which may promote aggressive tumor behavior [24]. However, the roles of PSC-derived miRNAs in the tumor microenvironment have not been elucidated.

PSCs play a pivotal role in the desmoplastic reaction, which promotes tumor development and inhibits drug penetration and uptake. Several autocrine and paracrine factors including miRNAs have been shown to regulate PSCs and fibrosis. Kwon *et al*. demonstrated that TGF-β-dependent loss of mir-29 enhanced PSC-mediated accumulation of stromal proteins [25]. Additionally, elevated expression of mir-130a and mir-221 was observed in serum from patients with early chronic pancreatitis, suggesting they may contribute to pancreatic fibrosis.

Let-7d has been associated with lung and liver fibrosis [26, 27]. We found that let-7d could target 18 genes in the TGF-β pathway. TGF-β can induce pancreatic fibrosis and is often upregulated in fibrotic tissue [28–30]. Our analysis of TCGA data indicated that the expression of 10 out of 18 genes in the TGF-β pathway was negatively correlated with let-7d expression (only THBS1 showed a significant difference). THBS1 activates latent TGF-β [31] and has been associated with pathological fibrosis of the kidney and muscle [32–34]. Modulation of these miRNAs may decrease PDAC-related pancreatic fibrosis and enhance the efficacy of anti-tumor agents.

Our study was limited by the relatively small number of samples that were collected at a single institution. These results also should be evaluated in future studies that focus on clarifying the molecular mechanism how miRNAs modulate fibrosis in PDAC. Our results indicate that fibrosis-related miRNAs can be useful serum diagnostic and/or prognostic biomarkers for pancreatic ductal adenocarcinoma.

## MATERIALS AND METHODS

### Patients and sample collection

We prospectively enrolled 45 PDAC patients and 42 controls in our study (Table 1). All PDAC patients underwent endoscopic ultrasound-guided fine-needle aspiration biopsy to confirm the diagnosis. The study protocol was approved by the Institutional Review Board of Fukushima Medical University. All participants provided written informed consent.

Patient clinical data, including age, sex, serum CEA levels, and serum CA 19-9 levels, were obtained from electronic medical records. The pathological characteristics of the tumors (e.g., size and location) were also retrieved. For serum collection, 8 mL of blood was collected and incubated at room temperature for at least 60 minutes to allow clotting. Samples were then centrifuged at 1,000 × g for 10 minutes. The serum was collected and stored in aliquots at –80°C.

Overall survival for PDAC patients treated with gemcitabine-based chemotherapy was calculated from the day of initiation of chemotherapy to either death or the date of the last follow-up examination.

### Analysis of RNA-Seq and miRNA-Seq data

The mRNA-Seq data for pancreatic tissue from 178 patients and miRNA-Seq data for 183 patients were obtained from TCGA (https://tcga-data.nci.nih.gov/tcga/). Both mRNA and miRNA data were available for 172 patients. Normalized mRNA and miRNA expression data (the calculated expression for all reads aligning to a particular mRNA per sample) were collected from TCGA Data Portal using the Subio platform version 1.21 (Kagoshima, Japan). Clinical data including prognosis and disease stage were also downloaded for each patient.

### Cell lines

Two commercially available PDAC cell lines (Panc-1 and BxPC-3) were obtained from the American Type Culture Collection. The immortalized hPSC line hPSC21-S/T was established by the introduction of the simian virus 40 T antigen and human telomerase reverse transcriptase into human primary PSCs [35]. The hPSCs expressed typical stellate cell markers including α-SMA, vimentin, COLA1, and glial fibrillary acidic protein [36]. The hPSCs were cultured in DMEM supplemented with 2 mM L-glutamine, 1 mM sodium pyruvate, 4.5 g/L glucose, 10% fetal bovine serum (FBS), and 1% penicillin-streptomycin. The other cell lines were grown in RPMI-1640 containing the same supplements. All cell lines were cultured in a humidified atmosphere containing 5% CO_2_ at 37°C and were grown to 70% to 80% confluence in 10 cm culture dishes prior to experiments.

### Co-culture experiments

Co-culture experiments were performed in 6-well plates with Corning Cell Culture Inserts (Corning, NY, USA; 0.45 μm pore size). PDAC cells (Panc-1 and BxPC-3) and hPSCs were seeded onto the culture inserts and plates, respectively, at a density of 5 × 10^5^ cells per well in 3 mL DMEM supplemented with 10% FBS. The cells were then cultured for 24 hours at 37°C. The cell culture inserts containing the PDAC cells were then placed into 6-well plates containing hPSCs and incubated for 72 hours. The inserts were then removed and the wells washed three times with phosphate-buffered saline (PBS) prior to analysis.

### Extraction of total RNA

Total RNA was extracted from cultured cells using the RNeasy Mini Kit (Qiagen, Hilden, Germany), and total RNA including miRNA was extracted using the miRNeasy Mini Kit (Qiagen) according to the manufacturer's protocols. The cDNA was prepared from 500 ng total RNA using the iScript™ Advanced cDNA Synthesis Kit (Bio-Rad, Hercules, CA, USA) and a TaqMan miRNA Reverse Transcription Kit (Thermo Fisher, Waltham, MA, USA) and primers to amplify mir-328-3p, mir-382-5p, mir-let-7d-5p, and mir-RNU48.

Total RNA was extracted from 200 μL of serum using the miRNeasy Serum/Plasma Kit (Qiagen) according to the manufacturer's protocol. Because low concentrations of total RNA were obtained from serum samples, 50 ng of total RNA was used to prepare cDNA for the serum miRNA analysis. Total RNA concentrations were measured at 260 nm (A260) using a NanoDrop (NanoDrop Technologies, Wilmington, DE, USA). The purity was determined by measuring the A260/A280 ratio. The A260/A280 values of all total RNA samples were > 1.8.

### Quantitative real-time PCR

One μL of cDNA was used as a template in a 20 μL PCR. PCR products were amplified using specific primers (TaqMan MiRNA Assay) and the TaqMan Universal PCR Master Mix II (Applied Biosystems, Foster City, CA, USA). PCR products were detected using the StepONE Plus Real Time PCR System (Applied Biosystems). Each sample was run in triplicate [23]. The following TaqMan probes for miRNA detection were obtained from Applied Biosystems: α-SMA (Hs00426835), PDGFRβ (Hs01019589), COL1A1 (Hs00164004), and GAPDH (Hs02786624) for cultured cells, and hsa-mir-328 (assay ID: 00543), hsa-mir-382 (assay ID: 000572), and hsa-mir-let-7d (assay ID: 002283) for miRNA analysis.

### MiRNA profiling and data validation

A pathway-focused miScript miRNA PCR Array (Qiagen) was used for miRNA profiling. The Fibrosis miRNA PCR Array (catalog number: MIHS-117Z) allowed the detection of 84 previously identified miRNAs, and included appropriate housekeeping assays and RNA quality controls. Assays were performed according to the manufacturer's protocol. Briefly, 500 ng of total RNA was extracted from cultured cells and reverse transcribed to generate cDNA using the miScript II RT Kit. Diluted cDNA was mixed with miScript Universal Primer and SYBR Green Master Mix, and then added to each well of a 96-well plate containing lyophilized primers. PCR was performed with a StepOnePlus Real-Time PCR System (Applied Biosystems). The expression of individual miRNAs was analyzed using the Ct values. Log two-fold changes in miRNA levels were calculated using the Cp values and the manufacturer's software. Fold changes in expression in Panc-1 and BxPC-3 cells co-cultured with hPSC compared to control hPSCs alone were calculated. Endogenous controls, real-time PCR negative and positive controls, and genomic DNA contamination controls were also analyzed for each array. Real-time PCR with TaqMan probes was performed to validate the results of the miRNA profiling.

### Transfection of hPSCs

For transient transfection, 5 × 10^5^ hPSCs were cultured in 6-well plates for 24 hours and then transfected using the Lipofectamine RNAiMAX Reagent (Invitrogen, Life Technologies). A total of 3 μL or 6 μL of 10 μM miRNA mimics (mirVana miRNA mimic for hsa-mir-328-3p and hsa-mir-382-5p, Thermo Fisher Scientific), inhibitor (mirVana miRNA inhibitor for hsa-mir-let-7d-5p), or negative controls were diluted in 150 μL serum-free media (the final concentrations were 10 nM and 20 nM, respectively), and then mixed with 9 μl Lipofectamine iRNAiMAX in 150 μL serum free media for 5 minutes at room temperature. These mimic/inhibitor-lipid complexes were added to the cells and incubated for 24 hours and 48 hours, respectively. Real-time PCR with TaqMan probes was performed to validate the results of the miRNA profiling.

### Pathway enrichment analysis

MiRNA targets were identified using the DIANA-miRPath v3.0 software (http://www.microrna.gr/miRPathv3) and experimentally validated miRNA interactions derived from DIANA-TarBase v7 [37].

### Digital PCR

Digital PCR and quantification of the absolute levels of serum miRNAs were performed using the Quant-Studio 3D Digital PCR System (Thermo Fisher Scientific). Data were analyzed using the QuantStudio 3D Analysis Suite Cloud Software (Thermo Fisher Scientific). The digital PCR mixture contained 5.0 μL of the RT product, 1.0 μL nuclease-free H_2_O, 7.50 μL of the QuantStudio™ 3D Digital PCR Master Mix, and 0.75 μL of the TaqMan MiRNA Assay-1 (20X) for let-7d [18]. Samples were individually loaded onto the QuantStudio 3D digital PCR 20K chip kit v2 using the QuantStudio 3D digital PCR Chip Loader. Digital PCR was performed in a Proflex 2× flat block thermal cycler (Applied Biosystems) using standard conditions: 96°C for 10 min followed by 39 cycles of 60°C for 2 min, 98°C for 30 sec, and 60°C for 2 min. Chips were read on the QuantStudio 3D Digital PCR instrument and the number of FAM-positive and FAM-negative (empty) wells quantified [20].

### Western blot analysis

Cells were washed twice with PBS and scraped from the plates in RIPA buffer (50 mM Tris HCl pH 8, 150 mM NaCl, 1% NP-40, 0.5% sodium deoxycholate, and 0.1% SDS) containing a Protease Inhibitor Cocktail (Thermo Fisher Scientific). Proteins were electrophoresed in sample buffer on acrylamide gels and then transferred to polyvinylidene difluoride membranes (Clear Blot Membrane-P; ATTO Co., Ltd., Tokyo, Japan). Membranes were blocked with 0.5% TBST containing 3% non-fat milk, incubated with the primary antibodies (1:1000) overnight at 4°C, and then incubated with horseradish peroxidase-conjugated anti-rabbit antibodies (1:3000; Sigma-Aldrich, St. Louis, MO, USA). Blots were visualized using the ECL Western Blotting Detection System (GE Healthcare, Buckinghamshire, UK). β-actin was used as a loading control. Antibodies against α-SMA, COL1A1, PDGFRβ, and β-actin were purchased from Cell Signaling Technology (Boston, MA, USA).

### Analysis of intra-tumor fibrosis on CT

Intra-tumor fibrosis was estimated as described previously [38]. Pancreatic images were acquired before and after the administration of contrast. Contrast-enhanced CT scans were acquired at 40 seconds (arterial phase) and 70 seconds (portal-venous phase). Hounsfield unit (HU) measurements from a hypodense area of tumor and from the normal pancreas were made during each phase of CT. The HU measurements for the tumor and normal pancreas were used to estimate tumor fibrosis using the following equations:

### Pancreatic cancer

HUmL = HU of the pancreatic mass at the arterial phase - HU of the pancreatic mass at the precontrast phase

HUm^2^ = HU of the pancreatic mass at the portal venous phase – HU of the pancreatic mass at the precontrast phase

AUC of the pancreatic mass = 5 * HUmL + 35 * (HUmL + HUm^2^)

### Normal pancreas

HUp1 = HU of the normal pancreas at the arterial phase - HU of the normal pancreas at the precontrast phase

HUp2 = HU of the normal pancreas at the portal venous phase – HU of the normal pancreas at the precontrast phase

AUC of the normal pancreas = 5 * HUp1 + 35 * (HUp1 + HUp2)

Normalized AUC = AUC of the pancreatic mass / AUC of the normal pancreas

### Statistical analysis

Continuous variables (i.e., age, tumor size, and serum CEA, CA 19-9 levels) are reported as the median value and range, and were compared using Mann-Whitney *U* tests. Gender was compared using Fisher's exact tests. The diagnostic accuracy of let-7d, CEA, and CA 19-9 levels was assessed using ROC curves (AUC). Correlations between intra-tumor fibrosis and serum miRNA levels were evaluated using Pearson's correlation analyses. Survival analysis was performed using the Kaplan-Meier method and log-rank tests. All statistical analysis was performed with GraphPad Prism 7.0 (GraphPad, San Diego, CA, USA). A *P* < 0.05 was considered statistically significant.

## SUPPLEMENTARY MATERIALS FIGURE



## Abbreviations

PCR: polymerase chain reaction
miRNA: microRNA
PDAC: pancreatic ductal adenocarcinoma
AUC: area under curve
PSC: pancreatic stellate cell
CEA: carcinoembryonic antigen
CA 19-9: carbohydrate antigen 19-9
α-SMA: alpha-smooth muscle actin
PDGFRβ: platelet derived growth factor receptor β
COL1A1: type I collagen.
