# Supplementary material for: Fibrosis-related miRNAs as serum biomarkers for pancreatic ductal adenocarcinoma
Source: Oncotarget. 2017 Dec 17;9(4):4451–60. doi: 10.18632/oncotarget.23377 (PMC5796986; doi:10.18632/oncotarget.23377)
Supplement: Supplementary file 1 [file oncotarget-09-4451-s001.pdf]

Fibrosis-related miRNAs as serum biomarkers for pancreatic ductal adenocarcinoma

SUPPLEMENTARY MATERIALS

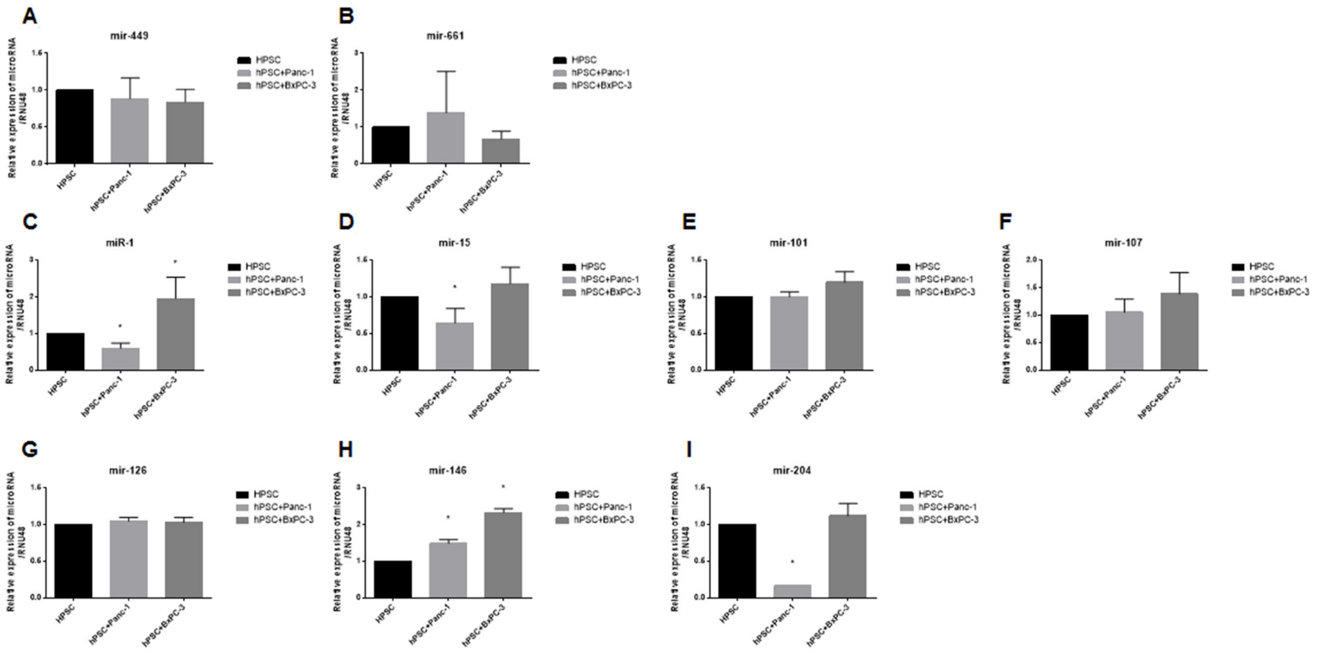

Supplementary Figure 1: MicroRNA validation studies with Taqman probes. The expression of each miRNA did not increase (**A–B**) or decrease (**C–I**) as observed in miRNA profiling assays. $^{*}P < 0.05$.
